# Supplementary material for: Multi-scale structures of the mammalian radial spoke and divergence of axonemal complexes in ependymal cilia
Source: Nat Commun. 2024 Jan 8;15:362. doi: 10.1038/s41467-023-44577-1 (PMC10774353; doi:10.1038/s41467-023-44577-1)
Supplement: Supplementary file 1 — Supplementary Information [file 41467_2023_44577_MOESM1_ESM.pdf]

Supplementary information

Multi-scale structures of the mammalian radial spoke and divergence of axonemal complexes in ependymal cilia

Xueming Meng[1,#], Cong Xu[1,#], Jiawei Li[1,#], Benhua Qiu[2,#], Jiajun Luo[2,#], Qin Hong[1], Yujie Tong[1], Chuyu Fang[2], Yanyan Feng[3], Rui Ma[4], Xiangyi Shi[4], Cheng Lin[1], Chen Pan[5], Xueliang Zhu[2,6,*], Xiumin Yan[3,*], Yao Cong[1,6,*]

[1] Key Laboratory of RNA Science and Engineering, Shanghai Institute of Biochemistry and Cell Biology, Center for Excellence in Molecular Cell Science, Chinese Academy of Sciences, Shanghai, 200031, China; University of Chinese Academy of Sciences

[2] State Key Laboratory of Cell Biology, Shanghai Institute of Biochemistry and Cell Biology, Center for Excellence in Molecular Cell Science, Chinese Academy of Sciences, Shanghai, China; University of Chinese Academy of Sciences

[3] Ministry of Education-Shanghai Key Laboratory of Children's Environmental Health, Institute of Early Life Health, Xinhua Hospital, Shanghai Jiao Tong University School of Medicine, Shanghai 200092, China

[4] Shanghai Nanoport, Thermofisher Scientific, Shanghai, China

[5] National Facility for Protein Science in Shanghai, Shanghai Advanced Research Institute, Chinese Academy of Sciences, Shanghai 201210, China

[6] Key Laboratory of Systems Health Science of Zhejiang Province, School of Life Science, Hangzhou Institute for Advanced Study, University of Chinese Academy of Sciences, Hangzhou, China.

[#] These authors contributed equally.

* To whom correspondence may be addressed. Email: xlzhu@sibcb.ac.cn, yanx@shsmu.edu.cn, or cong@sibcb.ac.cn

a

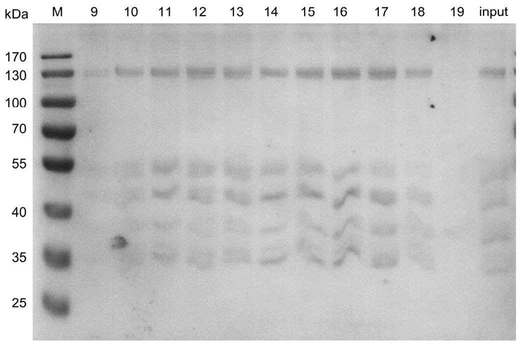

b

Subunit	Coverage [%]	PSMs	Unique Peptides
Rsph4a	69	211	43
Rsph3b	94	216	49
Rsph9	84	224	31
Rsph16	93	228	45
Rsph1	78	164	26
Rsph23	94	84	18
Rsph10b	60	88	57
Rsph2	71	61	17

c

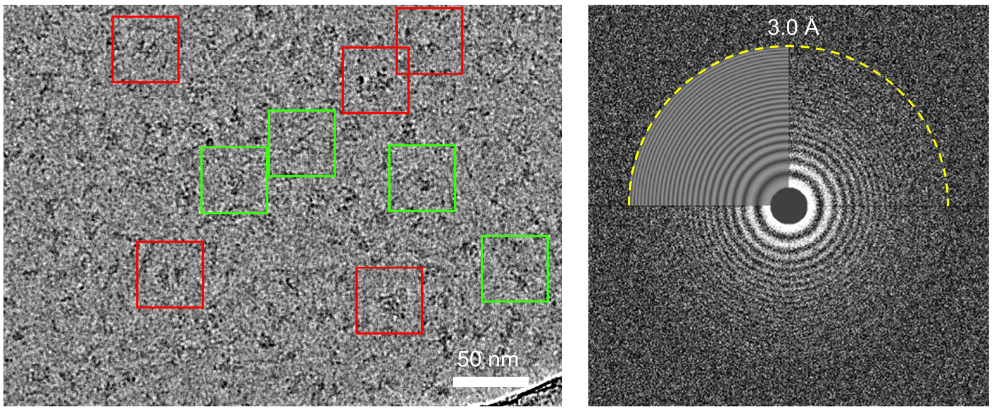

d

Constructions
pcDNA3.4-N-Strep II-Tev-Rsph1
pcDNA3.4-N-HA-Rsph2
pcDNA3.4-Rsph3b
pMLink-Rsph4a
pcDNA3.4-Rsph9
pcDNA3.4-N-3xFlag-Rsph16
pcDNA3.4-N-1xMyc-Rsph23
pcDNA3.4-N-Flag-Rsph3b
pcDNA3.1-Rsph10b
pLV-EGFP-AK8

Supplementary Fig. 1 The composition and cryo-EM analysis of the RS head-neck complex. a The RS head-neck complex was analyzed using a 10%-40% glycerol gradient ultra-centrifugation assay. Fractions were collected from top to bottom and subjected to SDS-PAGE analysis, followed by Coomassie Brilliant Blue staining. The results indicated that the RS head-neck complex was primarily distributed in fractions 10-18. This experiment was repeated three times independently with similar results. Source data are provided as a Source Data file. **b** Composition of the RS head-neck complex determined by mass spectrometry. **c** Representative cryo-EM micrograph (left), and its thon ring fitted to 3Å (right). Particles of RS head-neck monomer were marked in green and dimer in red rectangle. **d** Constructions of the RS head-neck complex and AK8.

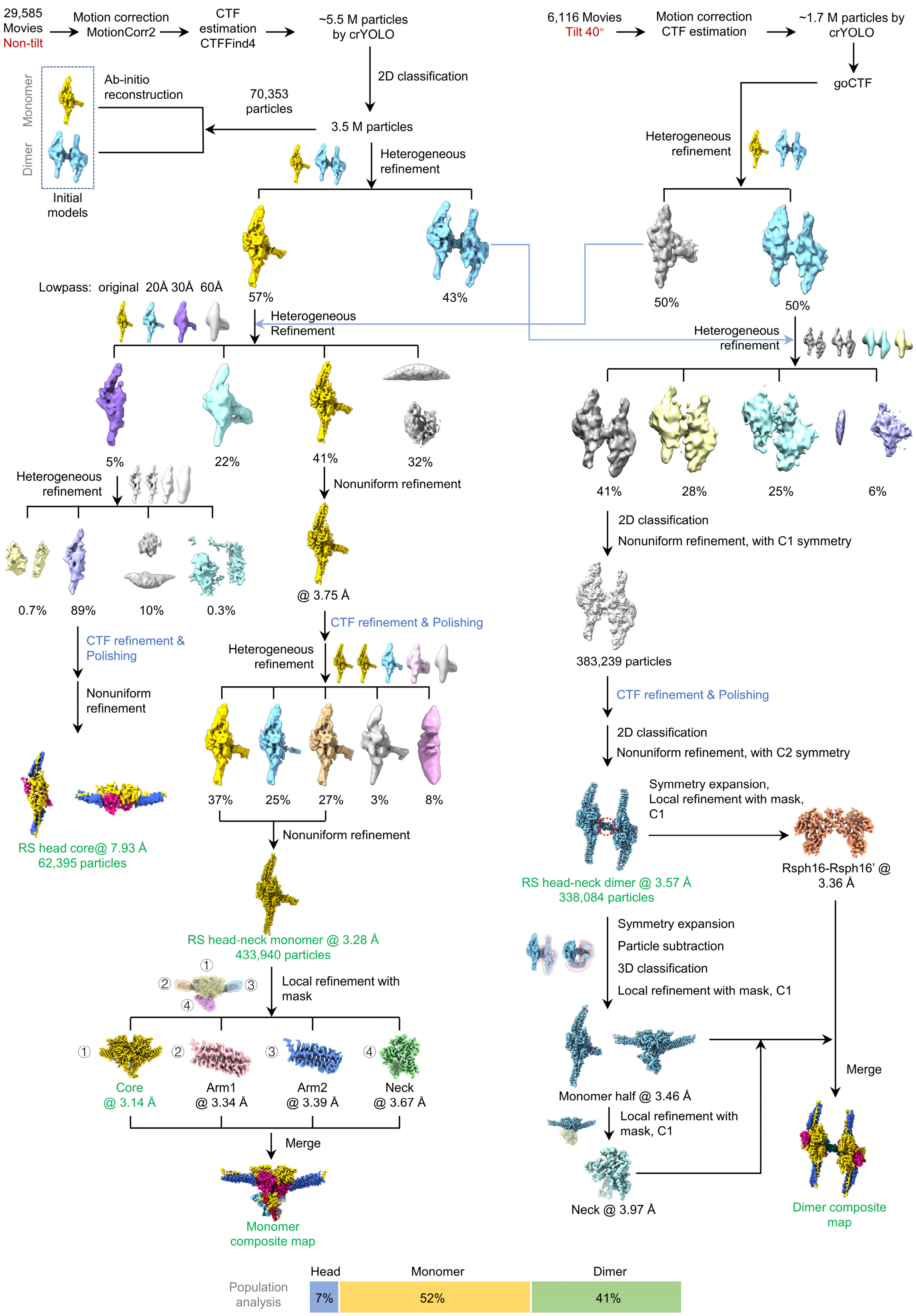

Supplementary Fig. 2 Cryo-EM data processing procedure and population analysis for the RS head-neck complex.

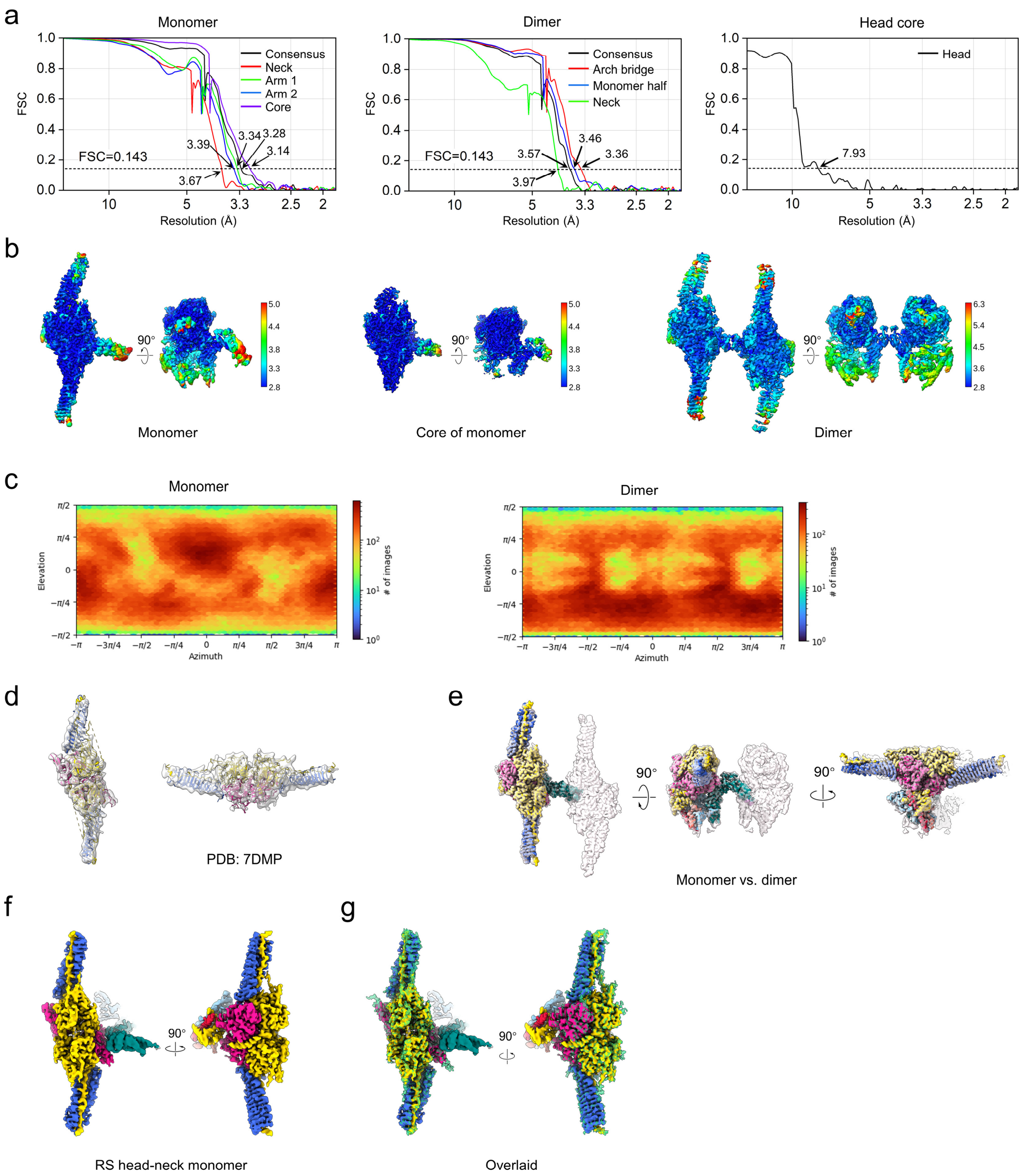

Supplementary Fig. 3 Assessment of the cryo-EM maps of RS head-neck complex. a Resolution estimation of the cryo-EM maps of the RS head-neck complex by the FSC at 0.143 criterion. **b** Local resolution evaluation for the RS head-neck monomer, monomer core, and dimer maps. **c** Angular distribution for the monomer and dimer maps. **d** The less populated RS head core structure resembles the one obtained in our previous study (PDB: 7DMP). **e** The good match between the monomer (in color) and dimer (transparent grey) maps in corresponding region, indicating no conformational variations when forming a dimer. **f** Our current RS head-neck monomer map visualized in the same angle as our previous RS head core map (EMDB: 30766) presented in Fig. 2C of Zheng et al[1]. **g** Overlaid structure comparison between the current RS head-neck monomer (in color) and the previous RS head core map (EMDB: 30766, in transparent green).

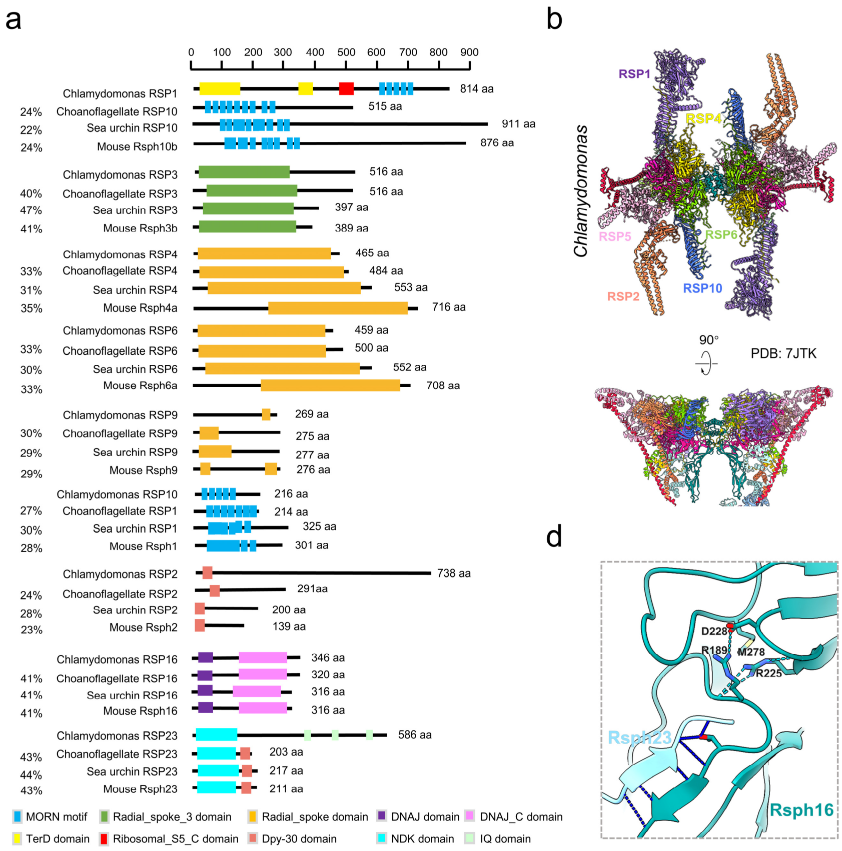

Supplementary Fig. 4 Compositional and conformational differences between mammalian and *Chlamydomonas* RS head-neck complex. a Domain diagrams of the RS head-neck subunits of *Chlamydomonas*, *Choanoflagellate*, Sea urchin, and *Mus musculus*. Sequence identity was compared between *Chlamydomonas* and its orthologues. **b** Different views of the *Chlamydomonas* RS head-neck dimer (PDB: 7JTK). **c** Overlaid cryo-EM maps of the *Chlamydomonas* (transparent, EMDB: 22475) and our mammalian RS head-neck dimer (in color). Their "arms" display conformational differences. **d** The M278R mutation in Rsph16 potentially disrupts the protein interaction networks between Rsph16 and Rsph23.

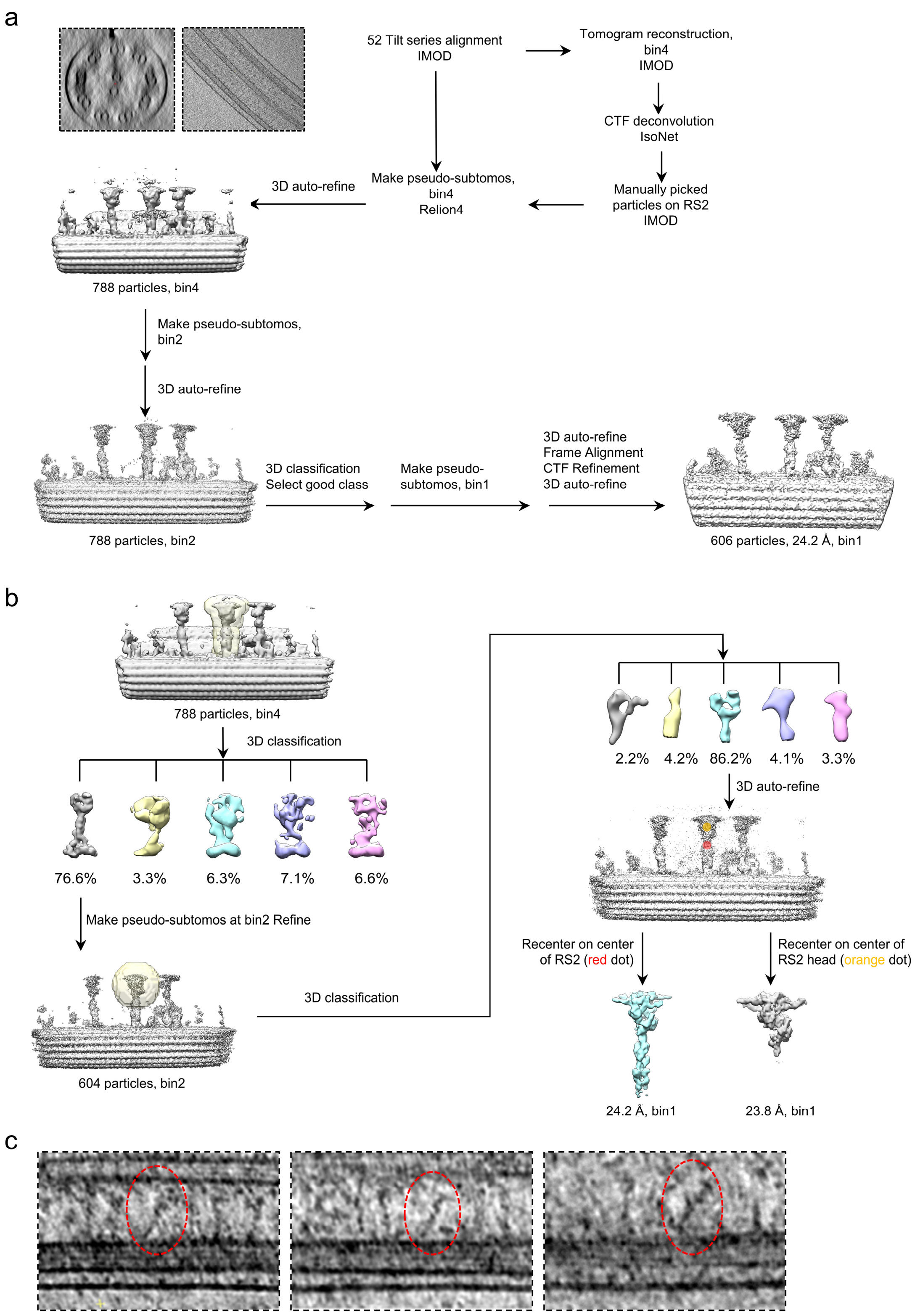

Supplementary Fig. 5 Cryo-ET data processing and sub-tomogram averaging procedures for mouse ependymal cilia. a Cryo-ET data processing procedure. **b** Focused 3D classification and refinement on RS2, as an example, to improve its resolution. **c** The occasional downward tilt of a complete RS1.

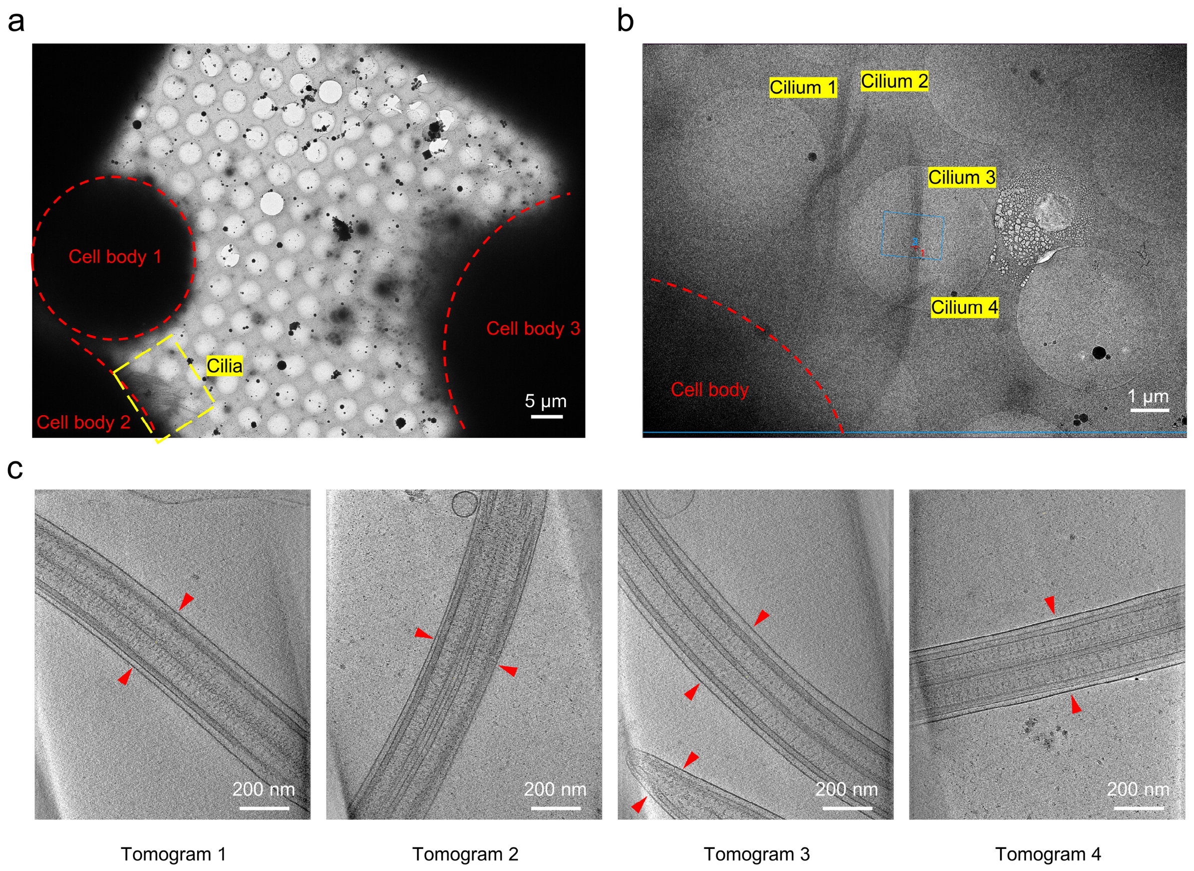

Supplementary Fig. 6 Quality of cilia preparations for cryo-ET imaging. a A representative search-mode image showing a bundle of cilia (yellow rectangle, bottom-left) connected to cell body (red circle). **b** A representative enlarged view showing four cilia connected to a cell body. **c** Four representative tomograms showing cilia with intact membrane (indicated by red arrowheads).

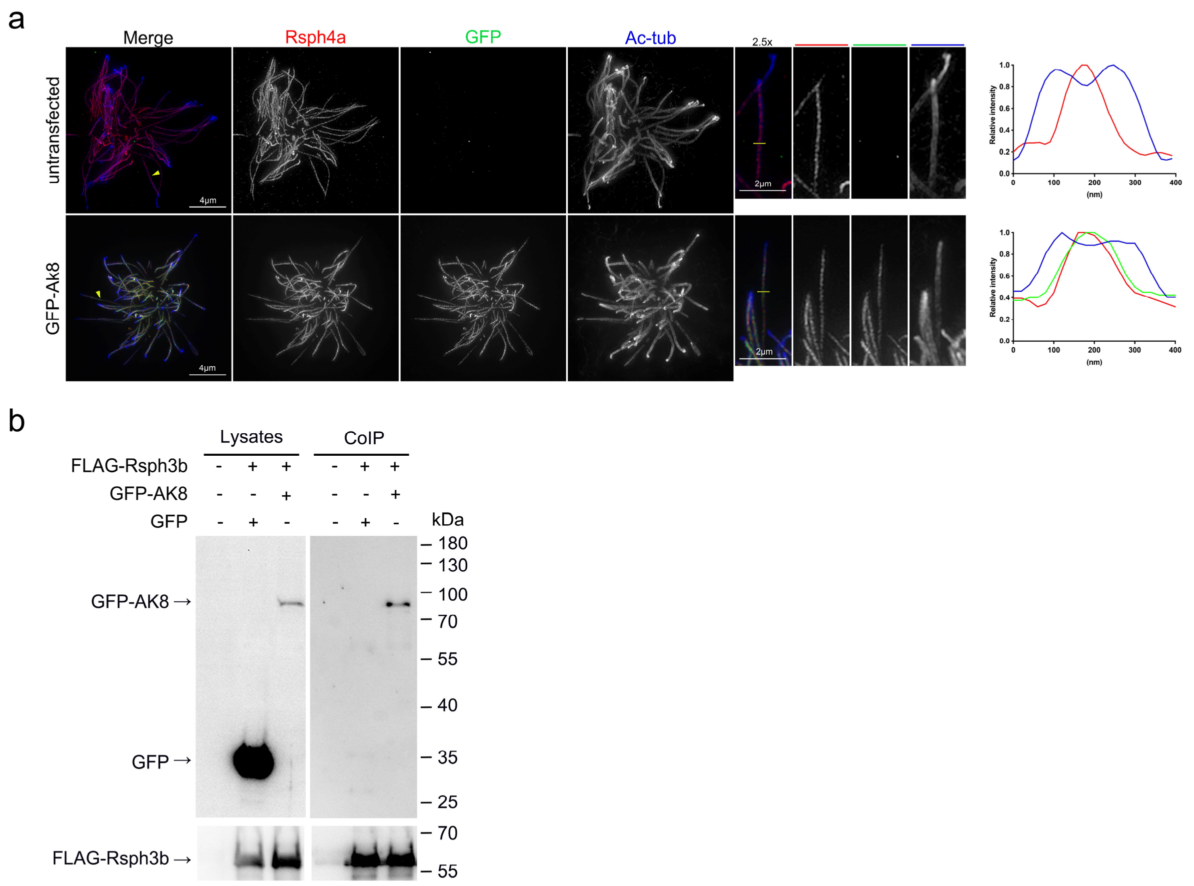

Supplementary Fig. 7 AK8 is a component of RS. a AK8 colocalized with Rsph4a in ependymal cilia. Cultured mEPCs were infected with lentivirus to express GFP-AK8 on one day before serum starvation (day -1) and fixed on day 10 for immunostaining and visualization using Hessian structured illumination microscopy. Acetylated tubulin (Ac-tub) marked axonemes. Cilia pointed by arrow heads were magnified by 2.5-fold to show details. Line scans were performed at positions marked by yellow lines to further show colocalization of GFP-AK8 with Rsph4a in the axonemal central lumen of cilia. **b** AK8 was associated with Rsph3b. HEK293T cells were transiently transfected to co-express FLAG-Rsph3b with either GFP-AK8 or GFP. Co-immunoprecipitation (CoIP) was performed using the cell lysates, followed by immunoblotting. Source data are provided as a Source Data file.

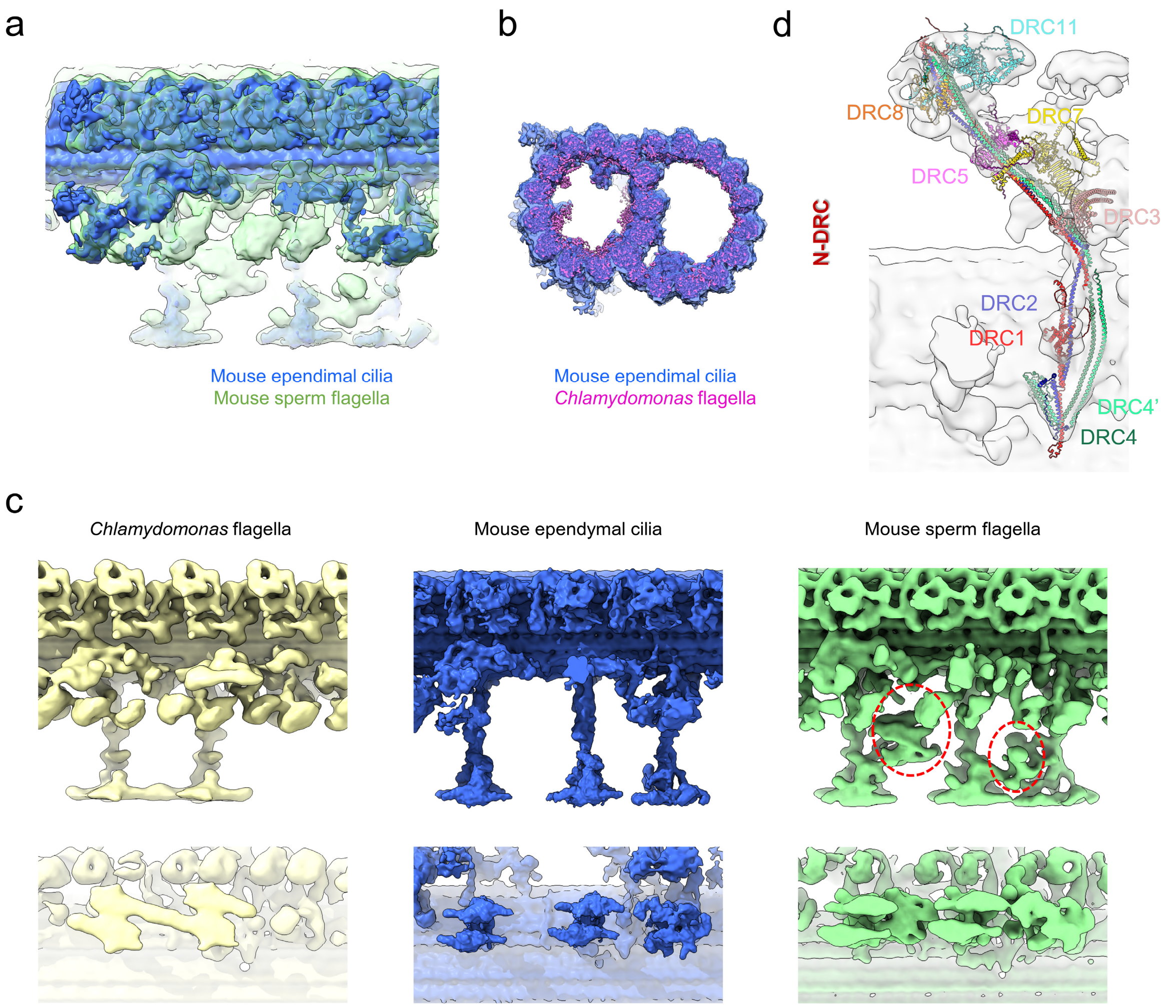

Supplementary Fig. 8 Comparation of RS and DMTs in mouse ependymal cilia with other species or tissues and the *in situ* N-DRC structure. a Overlaid cryo-ET maps of the mouse ependymal cilia (royal blue) and mouse sperm flagella (transparent green, EMD-27444). **b** Cross-section view of the overlaid cryo-ET maps of the ependymal cilia DMT (transparent royal blue) vs. the *Chlamydomonas* flagella DMT (magenta, EMD-9768). **c** Cryo-ET maps of 96 nm repeat for *Chlamydomonas* (kaki, EMD-9768), mouse ependymal cilia (royal blue, current study), and mouse sperm flagella (light green, EMD-27444) with the dotted red ellipsoid indicating sperm-specific components. **d** Proposed model fits into the N-DRC cryo-ET map of mouse ependymal cilia. The N-DRC in mouse ependymal cilia displays a strong contact in the upper left lobe of the V-shaped density with the B-tubule of the neighboring DMT, while the distal right lobe density is rather weak.

Supplementary Table 1 Cryo-EM data statistics of RS head-neck complex

	RS monomer	RS dimer	RS head
**Data collection**			
EM equipment	Titan Krios	Titan Krios	Titan Krios
Voltage (kV)	300	300	300
Detector	Gatan K3 camera	Gatan K3 camera	Gatan K3 camera
Magnification	81,000	81,000	81,000
Pixel size (Å)	0.854	0.854	0.854
Electron dose ($e^-/Å^2$)	54	54	54
Exposure time (s)	2.41	2.41	2.41
Frames	30	30	30
Defocus range (μm)	-1.0 to -2.7	-1.0 to -2.7	-1.0 to -2.7
**Reconstruction**			
Software		Relion 3.1& cryoSPARC	
Final particles	433,940	338,084	62,395
Symmetry	C1	C2	C1
FSC threshold	0.143	0.143	0.143
Final overall resolution (Å)	3.28	3.57	7.93
Resolution Range (Å)	2.8-6.0	3.0-7.0	NA
**Atomic modeling**			
Softwares		ChimeraX & Rosetta & Phenix & Coot & StarMap	
Model composition:			
Non hydrogen atoms	17,614	33,962	NA
Residues	2,182	4,208	NA
**Validation:**			
Bond length (Å)	0.005	0.008	NA
Bond Angle (°)	1.115	1.549	NA
Poor rotamers (%)	0.63	0.80	NA
Molprobity score	1.61	1.68	NA
Clash score	5.72	5.54	NA
**Ramachandran plot (%):**			
Favored	95.73	94.50	NA
Allowed	4.23	5.40	NA
Outliers	0.05	0.10	NA

Supplementary Table 2 Cryo-ET data statistics of mouse ependymal cilia

Maps	Resolution (Å)	Subtomo #
96-nm axonemal repeat with RS1/2/3	24.2	606
48-nm repeat DMT	19.6	1574
RS1 (RS1 head)	25.0 (25.0)	266 (324)
RS2 (RS2 head）	24.2 (23.8)	520
RS3 (RS3 head)	27.0 (28.9)	582
N-DRC	24.6	606
IDA-I1/fβ-I1/fα-a	25.4	604
IDA-g/d	24.0	582

Supplementary Table 3 XL-MS detected subunit interactions of RS head-neck complex*

Protein1(site)-protein2(site)	Peptides	Best E-value	Spec count
Rsph2(77) - Rsph9(199)	QEELQLKEAR(7)-NKTLLEK(2)	1.03E-10	3
Rsph1(177) - Rsph4a(411)	FMNKNPVGPGK(4)-SLYKAPQVIPK(4)	5.36E-09	3
Rsph4a(221) - Rsph9(36)	AYLLSTSSKSGLNLYDHLSK(7)-ASLLTSLMLVKR(11)	1.66E-13	3
Rsph16(167) - Rsph3b(251)	GVQKQDPPIER(4)-NETLQKISALIFAR(6)	3.99E-10	5
Rsph16(167) - Rsph3b(97)	GVQKQDPPIER(4)-AQEQLKPR(6)	1.93E-11	4
Rsph2(77) - Rsph4a(223)	RQEELQLKEAR(8)-AYLLSTSSKSGLNLYDHLSK(9)	4.00E-08	3
Rsph4a(220) - Rsph9(36)	AYLLSTSSKSGLNLYDHLSK(6)-ASLLTSLMLVKR(11)	1.36E-10	2
Rsph16(167) - Rsph10b(382)	GVQKQDPPIER(4)-KLDGSESR(1)	2.01E-09	3
Rsph16(167) - Rsph3b(272)	GVQKQDPPIER(4)-LDHKFDLMYAK(4)	1.34E-11	3
Rsph2(68) - Rsph9(199)	SITVAEEKR(8)-NKTLLEK(2)	1.49E-10	2
Rsph2(68) - Rsph9(161)	SITVAEEKR(8)-GALFKTPFGVTHVNR(5)	6.49E-09	8
Rsph16(98) - Rsph3b(97)	GIYDKFGEEGLK(5)-AQEQLKPR(6)	8.35E-06	3
Rsph2(68) - Rsph9(197)	SITVAEEKR(8)-EAIDLKNK(6)	2.50E-16	5
Rsph16(250) - Rsph3b(97)	EKLHPR(2)-AQEQLKPR(6)	2.64E-07	2
Rsph3b(251) - Rsph9(39)	NETLQKISALIFAR(6)-RDYRFAR(3)	3.86E-03	2
Rsph16(67) - Rsph3b(272)	NHPLKSSEPGAPEIFK(5)-QYLANLLPSVFDKLR(13)	4.52E-06	2
Rsph16(67) - Rsph3b(97)	NHPLKSSEPGAPEIFK(5)-AQEQLKPR(6)	1.36E-05	3
Rsph1(69) - Rsph4a(407)	HGQGTYKFK(5)-DNGGSEAGEEEEEELPKSLYK(17)	2.99E-09	2
Rsph16(300) - Rsph3b(97)	LLNIPINDIVHPKYFK(13)-AQEQLKPR(6)	2.42E-07	2
Rsph1(71) - Rsph4a(407)	HGQGTYKFK(7)-DNGGSEAGEEEEEELPKSLYK(17)	4.12E-12	2
Rsph1(184) - Rsph9(199)	NPVGPGKYVFDIGCEQHGEYR(7)-NKTLLEK(2)	2.85E-10	4

* We used best E-value (1.00E-02) and Spec count of at least 2 as the thresholds to remove extra XL-MS data with lower confidence.

Supplementary Table 4 RS head-neck subunit interaction networks*

Subunit	Residue	Atom	Subunit	Residue	Atom	Distance	Interaction
	TYR667	N		TYR128	OH	3.2	H-bond
	TYR670	N		TYR105	OH	3.51	H-bond
	ILE470	O		ASN156	ND2	2.8	H-bond
	GLU656	OE2		ARG187	NH2	2.78	H-bond
	TYR667	O		ARG122	NH2	2.81	H-bond
	GLN668	OE1		LYS166	NZ	3.71	H-bond
	GLU669	OE1		TYR107	OH	3.56	H-bond
	GLU669	OE1		ARG122	NE	2.92	H-bond
	GLU669	OE2		ARG99	NH2	2.45	H-bond
	GLU675	O		ASN63	ND2	3.03	H-bond
	GLU675	OE2		ASN63	ND2	2.42	H-bond
	GLU678	OE1		LYS76	NZ	2.84	H-bond
	GLU678	OE2		TYR71	OH	3.55	H-bond
	MET679	O		ARG53	NH2	2.8	H-bond
	ASP681	OD1		ARG30	NH1	3.85	H-bond
Rsph4a	ASP681	OD2	Rsph1	ASN74	ND2	3.9	H-bond
	PRO682	O		ARG30	NE	3.46	H-bond
	GLU687	OE2		ARG24	NH2	2.82	H-bond
	GLU656	OE2		ARG187	NH1	3.7	Salt bridge
	GLU656	OE2		ARG187	NH2	2.78	Salt bridge
	GLU669	OE1		ARG122	NE	2.92	Salt bridge
	GLU669	OE1		ARG122	NH2	3.07	Salt bridge
	GLU669	OE2		ARG99	NE	3.58	Salt bridge
	GLU669	OE2		ARG99	NH2	2.45	Salt bridge
	GLU678	OE1		LYS76	NZ	2.84	Salt bridge
	GLU678	OE2		LYS76	NZ	3.93	Salt bridge
	ASP681	OD1		ARG30	NE	3.97	Salt bridge
	ASP681	OD1		ARG30	NH1	3.85	Salt bridge
	GLU687	OE1		ARG24	NH2	3.37	Salt bridge
	GLU687	OE2		ARG24	NH1	3.26	Salt bridge
	GLU687	OE2		ARG24	NH2	2.82	Salt bridge
	TYR229	OH		ARG310	O	3.16	H-bond
	ASP230	OD1		ARG310	NH1	3.36	H-bond
Rsph4a	ASP230	OD1	Rsph3b	ARG310	NH2	3.79	H-bond
	ASP230	OD1		ARG310	NH1	3.36	Salt bridge
	ASP230	OD1		ARG310	NH2	3.79	Salt bridge
	GLY325	O		GLY19	N	2.6	H-bond
	GLU322	OE2		ARG25	NH2	3.82	H-bond
	ALA324	O		ARG49	NH1	3.82	H-bond
	GLY325	O		ARG49	NH2	3.43	H-bond
	TYR653	OH		ARG102	N	3.85	H-bond
	LEU361	O		SER227	OG	2.63	H-bond
	GLY360	O		SER227	OG	3.29	H-bond
	LEU361	O		ILE228	N	3.85	H-bond
	ASN364	OD1		GLN229	NE2	2.82	H-bond
	LYS357	NZ		GLY15	O	2.44	H-bond
	GLY327	N		GLY17	O	3.09	H-bond
	TYR634	OH		GLN18	OE1	2.92	H-bond
	ARG626	NH2		LEU51	O	3.06	H-bond
	ARG626	NH1		GLY52	O	2.98	H-bond
Rsph4a	THR609	OG1	Rsph9	LEU53	O	2.54	H-bond
	ARG626	NH2		ASP56	OD1	3.38	H-bond
	ASN364	ND2		GLU231	OE1	3.11	H-bond
	ALA279	N		ASN234	OD1	3.75	H-bond
	TYR653	OH		GLU267	OE2	2.33	H-bond
	LYS458	NZ		ASP271	OD2	3.19	H-bond
	LYS459	NZ		PHE274	O	3.4	H-bond
	PHE460	N		MET275	O	3.13	H-bond
	LYS458	NZ		MET275	SD	2.93	H-bond
	HIS651	NE2		MET275	SD	3.34	H-bond
	LYS459	NZ		LEU276	OXT	3.48	H-bond
	GLU322	OE2		ARG25	NH2	3.82	Salt bridge
	ASP331	OD2		ARG232	NH2	3.91	Salt bridge
	ARG626	NH2		ASP56	OD1	3.38	Salt bridge
	LYS458	NZ		ASP271	OD2	3.19	Salt bridge
	LYS459	NZ		LEU276	OXT	3.48	Salt bridge

(Continued 1)

Subunit	Residue	Atom	Subunit	Residue	Atom	Distance	Interaction
Rsph4a	MET260	O	Rsph2	HIS39	NE2	2.42	H-bond
	ALA261	O		HIS39	NE2	3.69	Salt bridge
	GLU273	N		LYS46	O	2.86	H-bond
	HIS271	O		HIS48	N	3.48	H-bond
	ASN272	OD1		ARG45	NE	2.59	H-bond
	GLU273	OE1		LYS46	NZ	3.87	H-bond
Rsph4a	SER219	OG	Rsph23	ILE41	O	3.41	H-bond
	THR220	N		ILE42	O	3.03	H-bond
	SER219	OG		ILE42	O	2.89	H-bond
	THR220	OG1		GLN43	NE2	3.53	H-bond
	LYS214	NZ		GLU149	OE2	3.55	Salt bridge
	GLU273	OE1		LYS46	NZ	3.87	Salt bridge
	TYR216	OH		ARG243	N	2.52	H-bond
	ASP246	OD1		SER221	OG	3.27	H-bond
	ASP246	OD2		LYS223	NZ	2.88	H-bond
	TYR216	OH		ILE239	O	3.12	H-bond
	TYR216	OH		ASP241	O	2.32	H-bond
	TYR216	OH		ARG243	O	3.06	H-bond
	ARG243	NH1		TYR216	O	2.57	H-bond
	SER255	OG		GLU252	OE2	3.77	H-bond
Rsph4a	LYS259	NZ	Rsph4a'	GLU252	OE2	3.59	H-bond
	LYS259	NZ		VAL248	O	3.21	H-bond
	GLU316	N		TYR319	OH	2.98	H-bond
	TYR620	OH		LEU311	O	2.4	H-bond
	TYR319	OH		GLU316	N	2.99	H-bond
	LEU311	O		TYR620	OH	2.94	H-bond
	ASP246	OD2		LYS223	NZ	2.88	Salt bridge
	ASP249	OD1		LYS259	NZ	3.1	Salt bridge
	LYS259	NZ		GLU252	OE2	3.59	Salt bridge
Rsph4a	LYS223	NZ	Rsph9'	GLU65	OE1	3.36	H-bond
	LYS223	NZ		GLU65	OE1	3.36	Salt bridge
	SER662	OG		ASN167	OD1	3.7	H-bond
	TYR667	N		TYR128	OH	2.75	H-bond
	MET679	N		TYR59	OH	3.47	H-bond
	GLU656	OE1		ARG187	NH2	3.24	H-bond
	ASN657	OD1		GLY194	N	2.85	H-bond
	TYR667	O		ARG122	NH1	3.03	H-bond
	TYR667	O		TYR128	OH	3.57	H-bond
	GLN668	OE1		ARG122	NH2	3.09	H-bond
	GLU669	OE2		TYR107	OH	3.52	H-bond
	GLU675	OE1		ASN63	ND2	2.42	H-bond
	GLU678	OE1		LYS76	NZ	3.57	H-bond
	GLU678	OE2		TYR71	OH	2.83	H-bond
	MET679	O		ARG53	NH1	2.42	H-bond
Rsph4a'	ASN680	O	Rsph1'	ASN74	ND2	3.12	H-bond
	ASN680	OD1		ARG53	NH2	3.62	H-bond
	ASN680	OD1		ASN74	ND2	3.52	H-bond
	ASP681	OD2		ASN74	ND2	3.7	H-bond
	PRO682	O		ARG30	NE	3.2	H-bond
	PRO682	O		ARG30	NH2	3.3	H-bond
	GLU687	OE1		ARG30	NH2	3.54	H-bond
	GLU656	OE1		ARG187	NH2	3.24	Salt bridge
	GLU669	OE1		ARG99	NH2	3.94	Salt bridge
	GLU669	OE2		ARG122	NE	3.74	Salt bridge
	GLU678	OE1		LYS76	NZ	3.57	Salt bridge
	GLU678	OE2		LYS76	NZ	3.71	Salt bridge
	ASP681	OD1		ARG30	NE	3.32	Salt bridge
	GLU687	OE1		ARG30	NH2	3.54	Salt bridge
	ARG243	NE		THR172	OG1	2.73	H-bond
	GLU242	OE1		ARG156	NH1	3.12	H-bond
	ASP257	OD1		ARG171	NH1	3.2	H-bond
Rsph4a'	GLU242	OE1	Rsph9	ARG156	NH2	3.28	Salt bridge
	GLU242	OE1		ARG156	NH1	3.12	Salt bridge
	GLU242	OE2		ARG156	NH2	3.63	Salt bridge
	ASP257	OD1		ARG171	NH1	3.2	Salt bridge

* Interaction networks were determined using the PDBePISA v1.52[2] online service.

(Continued 2)

Subunit	Residue	Atom	Subunit	Residue	Atom	Distance	Interaction
Rsph4a'	TYR229	OH	Rsph3b	ASP319	OD2	2.51	H-bond
	THR237	O		ARG324	NH1	3.77	H-bond
	THR237	OG1		ARG323	NH2	2.5	H-bond
Rsph4a'	ASP246	O	Rsph9'	ARG171	NH2	2.52	H-bond
	ASP246	OD1		ARG171	NH2	2.79	H-bond
	ASP246	OD1		ARG171	NH1	3.26	Salt bridge
	ASP246	OD1		ARG171	NH2	2.79	Salt bridge
	ASP246	OD2		ARG171	NH1	3.57	Salt bridge
Rsph9	LYS161	O	Rsph3b	TYR328	OH	2.56	H-bond
Rsph9	LYS183	NZ	Rsph23	ASP32	OD1	2.71	H-bond
	LYS183	NZ		ASP32	OD1	2.71	Salt bridge
Rsph9	ARG40	NH1	Rsph4a'	GLU273	OE2	3.64	H-bond
	ARG40	NH2		GLU273	OE2	3.27	H-bond
	ARG37	NH2		GLU284	OE2	2.81	H-bond
	LYS36	O		TYR280	OH	3.29	H-bond
	ARG40	NH1		GLU273	OE2	3.64	Salt bridge
	ARG40	NH2		GLU273	OE2	3.27	Salt bridge
	ARG37	NE		GLU284	OE1	3.4	Salt bridge
	ARG37	NH1		GLU284	OE1	3.41	Salt bridge
	ARG37	NH2		GLU284	OE1	3.18	Salt bridge
	ARG37	NE		GLU284	OE2	3.85	Salt bridge
	ARG37	NH2		GLU284	OE2	2.81	Salt bridge
Rsph9	ARG223	NE	Rsph1	ILE178	O	2.67	H-bond
	SER97	O		ARG193	NH1	3.25	H-bond
	MET270	O		GLN182	NE2	3.36	H-bond
Rsph9'	GLU174	OE1	Rsph9	ARG171	NH2	3.32	H-bond
	GLU174	OE1		ARG171	NH2	3.32	Salt bridge
	GLU174	OE1		ARG171	NE	3.69	Salt bridge
Rsph9'	ARG223	NE	Rsph1'	GLU200	OE1	3.64	H-bond
	ARG223	NE		GLU200	OE2	3.61	H-bond
	MET270	O		GLN182	NE2	2.9	H-bond
	ARG223	NE		GLU200	OE1	3.64	Salt bridge
	ARG223	NE		GLU200	OE2	3.61	Salt bridge
Rsph9'	ASN234	ND2	Rsph4a'	GLU275	O	3.87	H-bond
	ASN234	ND2		MET276	O	3.06	H-bond
	SER21	OG		GLN323	O	3.46	H-bond
	LEU20	N		GLY325	O	3.26	H-bond
	SER227	OG		GLY360	O	2.93	H-bond
	GLN229	NE2		GLY360	O	3.1	H-bond
	ILE228	N		LEU361	O	3.43	H-bond
	SER227	OG		LEU361	O	2.58	H-bond
	GLN229	NE2		LEU361	O	3.71	H-bond
	GLN229	NE2		ASN364	OD1	2.87	H-bond
	ARG102	NH1		ASN628	OD1	3.11	H-bond
	ARG102	NH2		ASN628	OD1	3.19	H-bond
	GLN18	NE2		TYR634	OH	2.48	H-bond
	ARG102	N		TYR653	OH	3.75	H-bond
	SER100	OG		GLU656	OE1	3.27	H-bond
	GLY17	O		GLY327	N	2.99	H-bond
	GLN229	OE1		ASN364	ND2	3	H-bond
	MET275	SD		LYS459	N	3.72	H-bond
	LEU276	O		LYS459	NZ	3.33	H-bond
	MET275	O		PHE460	N	3.42	H-bond
	GLY52	O		ARG626	NH1	3.04	H-bond
	GLY52	O		ARG626	NH2	2.85	H-bond
	GLU267	OE2		TYR653	OH	3.35	H-bond
	ASP271	OD1		TYR658	N	3.02	H-bond
	LEU276	O		LYS459	NZ	3.33	Salt bridge
	LEU276	OXT		LYS459	NZ	3.82	Salt bridge
Rsph9'	ARG37	NH1	Rsph4a	GLU284	OE2	2.98	H-bond
	THR30	OG1		GLN286	OE1	3.8	H-bond
	ASP23	OD2		TYR334	OH	2.39	H-bond
	ALA26	O		ARG335	NH2	3.62	H-bond
	THR30	OG1		ARG335	NH1	2.76	H-bond
	LYS36	O		TYR280	OH	3.12	H-bond
	THR80	O		LYS287	NZ	3.88	H-bond
	ARG37	NH1		GLU284	OE2	2.98	Salt bridge
	ARG37	NH2		GLU284	OE2	3.02	Salt bridge

(Continued 3)

Subunit	Residue	Atom	Subunit	Residue	Atom	Distance	Interaction
Rsph16	ARG185	NH1	Rsph2	GLU31	OE2	2.92	H-bond
	ARG185	NH2		GLU31	OE2	2.95	H-bond
	ARG185	NH1		GLU31	OE2	2.92	Salt bridge
	ARG185	NH2		GLU31	OE2	2.95	Salt bridge
Rsph16	ASP253	O	Rsph9'	ARG40	NH2	2.49	H-bond
	ASP254	O		ARG40	NH2	3.23	H-bond
	CYS244	SG		TYR75	OH	2.92	H-bond
	ASN258	O		LEU84	N	2.62	H-bond
	ASN262	OD1		SER108	N	3.41	H-bond
	ASN262	O		LYS135	NZ	2.74	H-bond
	LEU256	N		ASP38	O	3.69	H-bond
	ASN258	N		TYR39	OH	3.63	H-bond
	ASN262	ND2		TYR75	OH	3.54	H-bond
	THR246	OG1		GLU81	OE2	2.29	H-bond
	ASN258	ND2		TRP82	O	2.73	H-bond
	ASN262	ND2		GLU137	OE1	2.64	H-bond
Rsph16'	ARG301	NH1	Rsph9'	GLU112	OE2	2.54	H-bond
	LYS307	NZ		SER108	OG	3.28	H-bond
	ARG301	NH1		GLU112	OE2	2.54	Salt bridge
	ARG301	NH2		GLU112	OE2	2.99	Salt bridge
Rsph16'	LYS306	NZ	Rsph16	GLN312	O	2.45	H-bond
	CYS244	SG		LYS307	NZ	3.19	H-bond
	CYS244	O		ARG311	NH2	2.44	H-bond
	LYS306	NZ		THR316	OXT	3.51	Salt bridge
Rsph23	ILE10	N	Rsph3b	ASP314	OD2	3.72	H-bond
	LEU7	O		ARG318	NH2	3.82	H-bond
	PRO8	O		ARG318	NH2	3.47	H-bond
	PRO8	O		THR311	OG1	3.18	H-bond
Rsph23	HIS53	NE2	Rsph16	THR156	OG1	3.36	H-bond
	LYS199	NZ		ASP183	OD2	2.63	H-bond
	LYS201	N		THR195	O	3.58	H-bond
	LYS201	NZ		GLU197	OE1	2.46	H-bond
	CYS203	N		ARG193	O	3.29	H-bond
	LYS201	O		THR195	N	3.54	H-bond
	CYS203	O		ARG193	N	3.29	H-bond
	LYS199	NZ		ASP183	OD2	2.63	Salt bridge
	LYS201	NZ		GLU197	OE1	2.46	Salt bridge
Rsph23	LYS180	NZ	Rsph2	ASP2	OD1	3.63	H-bond
	LYS178	NZ		ASP2	OD2	3.84	H-bond
	TYR160	OH		VAL21	O	3.34	H-bond
	ARG140	NH1		ARG25	O	3.74	H-bond
	ASN198	ND2		ASP28	OD1	3.06	H-bond
	ASN196	ND2		ASP28	OD2	3.18	H-bond
	PRO181	O		TYR5	OH	3.58	H-bond
	LYS180	NZ		ASP2	OD1	3.63	Salt bridge
	LYS178	NZ		ASP2	OD2	3.84	Salt bridge

Supplementary Table 5 Reported PCD/asthenospermia-related mutations of the RS head-neck complex

Protein	AA change in *Homo sapiens*		Mapping site in *Mus musculus*	Disease	Reference
Rsph1	p.G92Afs*10		p.G92Afs*10	PCD: nRDS, bronchiectasis, chronic bronchitis, rhinosinusitis, otitis	3
Rsph1	p.E29*	p.G103D	p.G103D	PCD: nRDS, bronchiectasis, rhinosinusitis, otitis; subfertility	3
Rsph1	p.E29*	p.R122Sfs*22	p.R122Sfs*22	PCD: nRDS, COPD, bronchiectasis, rhinosinusitis, otitis	3
Rsph1	c.366-3C>A	p.K136Mfs*6	p.K136Mfs*6	PCD: bronchiectasis, rhinosinusitis, otitis; Asthenospermia	3
Rsph4a	p.A369P		p.A368P	PCD: OM, sinusitis, rhinorrhea, chronic cough, wheeze, bronchitis, rRI, situs solitus, bronchiectasis, nRDS	4, 5
Rsph4a	p.G464E		p.G463E	PCD: nRDS, sinusitis, rhinorrhea, OM, chronic cough, wheeze, bronchitis, rRI, situs solitus, bronchiectasis	4, 6
Rsph9	p.R156*		p.R156*	PCD: bronchiectasis, sinusitis, rhinorrhea, OM, chronic cough, wheeze, bronchitis, rRI, situs solitus	4
Rsph9	p.H251R		p.H251R	PCD: sinusitis, rhinorrhea, OM, chronic cough, wheeze, bronchitis, rRI, situs solitus	4
Rsph16	p.M278R		p.M278R	PCD: nRDS, bronchitis, bronchiectasis, rhinosinusitis, otitis, infertility	7
Rsph16	Heterozygous	p.M309I	p.M309I	Idiopathic asthenospermia	8
Rsph23	p.W191*		p.W191*	PCD: sinusitis, OM, pneumonia, bronchiectasis, hearing loss	9
Rsph23	p.I139Yfs*8		p.I139Yfs*8	PCD: chronic infection, inflammation	10

Abbreviations: AA, Amino acid; PCD, primary ciliary dyskinesia; COPD, chronic obstructive pulmonary disease; nRDS, neonatal respiratory distress syndrome; OM, otitis media; rRI, recurrent respiratory infections

Supplementary Table 6 Information of radial spoke genes and their homologs

Gene	Mouse	UniProt	NCBI	Chlamydomonas ortholog
Rsph1	Rsph1	Q8VIG3	NM_001364916.1	RSP1
Rsph3b	Rsph3b	Q9DA80	NM_001083945.1	RSP3
Rsph4a	Rsph4a	Q8BYM7	NM_001162957.1	RSP4
Rsph9	Rsph9	Q9D9V4	NM_029338.4	RSP9
Dnajb13	Rsph16	Q80Y75	NM_153527.3	RSP16
Dydc2	Rsph2	Q9D3X8	NM_001360608.2	RSP2
Nme5	Rsph23	Q99MH5	NM_080637.4	RSP23
Ropn1l	Rsph11	Q9EQ00	NM_145852.2	RSP11
Ppil6	Rsph12	Q9D6D8	NM_028430.2	RSP12
Rsph14	Rsph14	Q9D3W1	NM_001163534.1	RSP14
LRRC34	Rsph15	Q9DAM1	NM_027941.1	RSP15
Calm1	Rsph20	P0DP26	NM_009790.5	RSP20
Cyb5d1	Cyb5d1	Q5NCY3	NM_001045525.2	FAP198
Dynll2	Rsph22	Q9D0M5	NM_026556.4	LC8 (RSP22)
Iqub	Iqub	Q8CDK3	NM_172535.3	FAP253
Morn3	Morn3	Q8C5T4	NM_029112.2	FAP207
Ak8	AK8	Q32M07	NM_001033874	
Rsp10b	Rsph10b	E9PYQ0	XM_006504895.1	RSP10
Cfap61	Cfap61	Q8CEL2	NM_001369121.1	FAP61
Cfap91	Cfap91	Q8BRC6	NM_001081025.1	FAP91 (RSP18)
Cfap251	Cfap251	E9Q743	NM_001370840.1	FAP251
Lrrc23	LRRC23	O35125	NM_001302555.1	

Supplementary Table 7 Mass spectrometry analysis of AK8 in mouse testis

IP: Rsph3b

Subunit	Coverage	PSMs	Peptides	Unique Peptides
Rsph1	52	89	15	15
Rsph2	60	23	11	11
Rsph3b	54	142	23	23
Rsph6a	32	147	18	18
Rsph9	46	117	15	15
Rsph16	56	96	20	20
Rsph23	43	24	8	8
AK8	65	231	43	43

IP: AK8

Subunit	Coverage	PSMs	Peptides	Unique Peptides
Rsph1	43	11	8	8
Rsph3b	19	5	5	4
Rsph6a	21	12	10	8
Rsph9	45	10	9	7
Rsph16	18	5	4	4
Rsph23	14	1	1	1
AK8	28	14	12	12

Supplementary References:

1. Zheng W*, et al.* Distinct architecture and composition of mouse axonemal radial spoke head revealed by cryo-EM. *Proc Natl Acad Sci U S A* **118**, (2021).

2. Krissinel E, Henrick K. Inference of macromolecular assemblies from crystalline state. *J Mol Biol* **372**, 774-797 (2007).

3. Kott E*, et al.* Loss-of-function mutations in RSPH1 cause primary ciliary dyskinesia with central-complex and radial-spoke defects. *Am J Hum Genet* **93**, 561-570 (2013).

4. Frommer A*, et al.* Immunofluorescence Analysis and Diagnosis of Primary Ciliary Dyskinesia with Radial Spoke Defects. *Am J Respir Cell Mol Biol* **53**, 563-573 (2015).

5. Emiralioglu N*, et al.* Genotype and phenotype evaluation of patients with primary ciliary dyskinesia: First results from Turkey. *Pediatr Pulmonol* **55**, 383-393 (2020).

6. Boaretto F*, et al.* Diagnosis of Primary Ciliary Dyskinesia by a Targeted Next-Generation Sequencing Panel: Molecular and Clinical Findings in Italian Patients. *J Mol Diagn* **18**, 912-922 (2016).

7. El Khouri E*, et al.* Mutations in DNAJB13, Encoding an HSP40 Family Member, Cause Primary Ciliary Dyskinesia and Male Infertility. *Am J Hum Genet* **99**, 489-500 (2016).

8. Li WN, Zhu L, Jia MM, Yin SL, Lu GX, Liu G. Missense mutation in *DNAJB13* gene correlated with male fertility in asthenozoospermia. *Andrology* **8**, 299-306 (2020).

9. Cho EH*, et al.* A nonsense variant in NME5 causes human primary ciliary dyskinesia with radial spoke defects. *Clin Genet* **98**, 64-68 (2020).

10. Sahabian A*, et al.* Generation of two hiPSC clones (MHHi019-A, MHHi019-B) from a primary ciliary dyskinesia patient carrying a homozygous deletion in the NME5 gene (c.415delA (p.Ile139Tyrfs*8)). *Stem Cell Res* **48**, 101988 (2020).
