# Supplementary material for: Multi-scale structures of the mammalian radial spoke and divergence of axonemal complexes in ependymal cilia
Source: Nat Commun. 2024 Jan 8;15:362. doi: 10.1038/s41467-023-44577-1 (PMC10774353; doi:10.1038/s41467-023-44577-1)
Supplement: Supplementary file 3 — Description of Additional Supplementary Files [file 41467_2023_44577_MOESM3_ESM.pdf]

File name: Supplementary Data 1

Description: Primer sequences.

File name: Supplementary Movie 1

Description: Cryo-EM map and model of the mammalian RS head-neck complex. The cryo-EM map is colored following the color schema as in Fig. 1. We also rendered the model in ribbon with individual subunit being labeled.

File name: Supplementary Movie 2

Description: Mode1 of the intrinsic motion of the RS head-neck dimer. This mainly displays an open-and-close movement between the Rsph1 arms of the two monomers within the dimer.

File name: Supplementary Movie 3

Description: Mode2 of the intrinsic motion of the RS head-neck dimer. This mainly exhibits a seesaw-like alternating up-and-down motions between the two monomers.

File name: Supplementary Movie 4

Description: Cryo-ET map of the 96-nm axonemal repeat of mouse ependymal cilia. RS1/RS2/RS3 are shown in distinct color.
