# Supplementary material for: Multi-scale structures of the mammalian radial spoke and divergence of axonemal complexes in ependymal cilia
Source: Nat Commun. 2024 Jan 8;15:362. doi: 10.1038/s41467-023-44577-1 (PMC10774353; doi:10.1038/s41467-023-44577-1)
Supplement: Supplementary file 9 — Reporting Summary [file 41467_2023_44577_MOESM9_ESM.pdf]

nature portfolio

Corresponding author(s): Xueliang Zhu, Xiumin Yan, Yao Cong

Last updated by author(s): Dec 6, 2023

Reporting Summary

Nature Portfolio wishes to improve the reproducibility of the work that we publish. This form provides structure for consistency and transparency in reporting. For further information on Nature Portfolio policies, see our Editorial Policies and the Editorial Policy Checklist.

Statistics

For all statistical analyses, confirm that the following items are present in the figure legend, table legend, main text, or Methods section.

n/a	Confirmed	
☐	☒	The exact sample size (n) for each experimental group/condition, given as a discrete number and unit of measurement
☐	☒	A statement on whether measurements were taken from distinct samples or whether the same sample was measured repeatedly
☒	☐	The statistical test(s) used AND whether they are one- or two-sided<br>*Only common tests should be described solely by name; describe more complex techniques in the Methods section.*
☒	☐	A description of all covariates tested
☒	☐	A description of any assumptions or corrections, such as tests of normality and adjustment for multiple comparisons
☒	☐	A full description of the statistical parameters including central tendency (e.g. means) or other basic estimates (e.g. regression coefficient) AND variation (e.g. standard deviation) or associated estimates of uncertainty (e.g. confidence intervals)
☒	☐	For null hypothesis testing, the test statistic (e.g. F, t, r) with confidence intervals, effect sizes, degrees of freedom and P value noted<br>*Give P values as exact values whenever suitable.*
☒	☐	For Bayesian analysis, information on the choice of priors and Markov chain Monte Carlo settings
☒	☐	For hierarchical and complex designs, identification of the appropriate level for tests and full reporting of outcomes
☒	☐	Estimates of effect sizes (e.g. Cohen's d, Pearson's r), indicating how they were calculated

Our web collection on statistics for biologists contains articles on many of the points above.

Software and code

Policy information about availability of computer code

Data collection	EPU 2.11, SreialEM 3.8
Data analysis	MotionCor2, RELION 3.1, CTFFIND 4.1.8, crYOLO 1.8.0, goCTF 1.1.0, StarMap 1.2.15, Phenix 1.19.2-4158, Rosetta 2017, COOT 0.9.7, UCSF Chimera 1.15, UCSF ChimeraX 1.5, PDBePISA v1.52, cryoSPARC v4, deepEMhancer 0.14, Warp 1.0.9 , IMOD 4.11, IsoNet 0.2.1, PEET 1.15.1, Relion 4.0, pLink2

For manuscripts utilizing custom algorithms or software that are central to the research but not yet described in published literature, software must be made available to editors and reviewers. We strongly encourage code deposition in a community repository (e.g. GitHub). See the Nature Portfolio guidelines for submitting code & software for further information.

Data

Policy information about availability of data

All manuscripts must include a data availability statement. This statement should provide the following information, where applicable:

- Accession codes, unique identifiers, or web links for publicly available datasets
- A description of any restrictions on data availability
- For clinical datasets or third party data, please ensure that the statement adheres to our policy

All data needed to evaluate the conclusions in the paper are present in the paper and/or the Supplementary Materials. Cryo-EM maps determined for the RS head-neck complex in monomer and dimer forms have been deposited at the Electron Microscopy Data Bank with accession numbers of EMD-37949 [https://

www.ebi.ac.uk/emdb/37949] (monomer composite map), EMD-38004 [https://www.ebi.ac.uk/emdb/38004] (monomer consensus map), EMD-38028 [https://www.ebi.ac.uk/emdb/38028] (monomer core), EMD-38029 [https://www.ebi.ac.uk/emdb/38029] (monomer arm 1), EMD-38030 [https://www.ebi.ac.uk/emdb/38030] (monomer arm 2), EMD-38031 [https://www.ebi.ac.uk/emdb/38031] (monomer neck) and EMD-38020 [https://www.ebi.ac.uk/emdb/38020] (dimer composite map), EMD-38003 [https://www.ebi.ac.uk/emdb/38003] (dimer consensus map), EMD-38013 [https://www.ebi.ac.uk/emdb/38013] (Rsph16-Rsph16' bridge of dimer), EMD-38014 [https://www.ebi.ac.uk/emdb/38014] (half of dimer), EMD-38019 [https://www.ebi.ac.uk/emdb/38019] (neck of dimer), and the associated atomic models have been deposited in the Protein Data Bank with accession numbers of 8WZB [http://doi.org/10.2210/pdb8WZB/pdb] (monomer) and 8X2U [http://doi.org/10.2210/pdb8X2U/pdb] (dimer). The mouse ependymal cilia cryo-ET maps generated in this study have been deposited in EMDB under the following accession numbers: EMD-37104 [https://www.ebi.ac.uk/emdb/37104] (96-nm repeat DMT), EMD-37111 [https://www.ebi.ac.uk/emdb/37111] (48-nm repeat DMT), EMD-37114 [https://www.ebi.ac.uk/emdb/37114] (RS1), EMD-37116 [https://www.ebi.ac.uk/emdb/37116] (RS1 head), EMD-37117 [https://www.ebi.ac.uk/emdb/37117] (RS2), EMD-37118 [https://www.ebi.ac.uk/emdb/37118] (RS2 head), EMD-37119 [https://www.ebi.ac.uk/emdb/37119] (RS3), EMD-37120 [https://www.ebi.ac.uk/emdb/37120] (RS3 head). Source data are provided with this paper.

Research involving human participants, their data, or biological material

Policy information about studies with human participants or human data. See also policy information about sex, gender (identity/presentation), and sexual orientation and race, ethnicity and racism.

Reporting on sex and gender	N/A
Reporting on race, ethnicity, or other socially relevant groupings	N/A
Population characteristics	N/A
Recruitment	N/A
Ethics oversight	N/A

Note that full information on the approval of the study protocol must also be provided in the manuscript.

Field-specific reporting

Please select the one below that is the best fit for your research. If you are not sure, read the appropriate sections before making your selection.

☒ Life sciences ☐ Behavioural & social sciences ☐ Ecological, evolutionary & environmental sciences

For a reference copy of the document with all sections, see nature.com/documents/nr-reporting-summary-flat.pdf

Life sciences study design

All studies must disclose on these points even when the disclosure is negative.

Sample size	No statistical methods were used to predetermine the sample size for cryo-EM and cryo-ET data because it is challenging to determine the number of micrographs or tomograms required before the data processing progresses. As a rule of thumb, several thousand images for cryo-EM and tens of tomograms for cryo-ET were used for the initial reconstruction. Additional images and tomograms were collected until a sufficient number was obtained to solve the structures with the resolution required for addressing specific scientific questions.
Data exclusions	The cryo-EM images of poor quality were deleted based on the defocus, astigmatism and resolution for better resolution. For cryo-ET data, images of poor quality were discarded and tilt series showed distorted ultrastructure of cilia were discarded.
Replication	Number of replicates stated in the figure legends where applicable.
Randomization	Randomization was not relevant for this study, as data were collected automatically and there were no samples allocated into control and experimental groups.
Blinding	Blinding was not relevant for this study, as data were collected automatically, and the data under investigation need to be known.

Reporting for specific materials, systems and methods

We require information from authors about some types of materials, experimental systems and methods used in many studies. Here, indicate whether each material, system or method listed is relevant to your study. If you are not sure if a list item applies to your research, read the appropriate section before selecting a response.

Materials & experimental systems

n/a	Involved in the study
☐	☒ Antibodies
☐	☒ Eukaryotic cell lines
☒	☐ Palaeontology and archaeology
☐	☒ Animals and other organisms
☒	☐ Clinical data
☒	☐ Dual use research of concern
☒	☐ Plants

Methods

n/a	Involved in the study
☒	☐ ChIP-seq
☒	☐ Flow cytometry
☒	☐ MRI-based neuroimaging

Antibodies

Antibodies used	RSPH3 polyclonal antibody(proteintech,17603-1-AP), https://www.ptgcn.com/products/RSPH3-Antibody-17603-1-AP.htm; Anti-AK8 antibody(Sigma, HPA021445-100ul), https://www.sigmaaldrich.cn/CN/zh/product/sigma/hpa021445; Rabbit IgG Isotype Control (Invitrogen,10500C), https://www.thermofisher.cn/cn/zh/antibody/product/Rabbit-IgG-Isotype-Control/10500C; mouse anti-acetylated tubulin (1:1000, Sigma-Aldrich, T6793), https://www.sigmaaldrich.cn/CN/zh/product/sigma/t6793; rabbit anti-Rsph4a (1:500, home made); anti-GFP (rabbit, MBL, Cat. # 598),598; and anti-FLAG (mouse, Sigma M2 F3165 lot. SLBN8915V), https://www.sigmaaldrich.cn/CN/zh/product/sigma/f3165;<br><br>Secondary antibodies used: goat-anti-rabbit Alexa Fluor546 (1:1000, Life Technologies, A-11035), goat-anti-mouse Alexa Fluor647 (1:1000, Life Technologies, A-31571); Anti-Mouse IgG(H+L)-HRP(goat, Life Technologies, G-21040), Anti-Rabbit IgG(H+L)-HRP(goat, Life Technologies, G-21213)
Validation	RSPH3 polyclonal antibody and Anti-AK8 antibody were applied in IPID MS, and significant level of relative components were detected while Rabbit IgG Isotype Control severed as negative control; mouse anti-acetylated tubulin antibody significantly show signal along the axoneme of cilia; rabbit anti-Rsph4a also showed signals along the axoneme of mEPC cilia, followed the distribution of radial spokes; anti-FLAG and anti-GFP antibodies both showed real positive signal at corresponding position in the gel blotting.

Eukaryotic cell lines

Policy information about cell lines and Sex and Gender in Research

Cell line source(s)	HEK293F suspension cells, Gibco, Catalog Numbers A14527; HEK293T cells (ATCC);The ependymal cells were isolated from C57BL/6J mice and cultured by prof. Xueliang Zhu's lab
Authentication	HEK293F suspension cells and HEK293T cells (ATCC) were not authenticated further after purchase. Mouse ependymal cells were checked by phase contrast microscopy to verify the growth of cilia.
Mycoplasma contamination	Cell lines have not recently been tested for Mycoplasma contamination.
Commonly misidentified lines (See ICLAC register)	No commonly misidentified lines were used.

Animals and other research organisms

Policy information about studies involving animals; ARRIVE guidelines recommended for reporting animal research, and Sex and Gender in Research

Laboratory animals	Male or female wild-type C57BL/6J mice of postnatal day 0 or 8-week-old were obtained from Shanghai SLAC Laboratory Animal for primary mouse ependymal cell culture or immunoprecipitation, respectively.
Wild animals	No wild animals were used.
Reporting on sex	Both male and female were used for cell culture with no bias for primary cell culture. Only male were used for Co-immunoprecipitation of Rsph3b or Ak8 in testes.
Field-collected samples	No field-collected samples were used.
Ethics oversight	All animal experiments were performed following guidelines approved by the Institutional Animal Care and Use Committee of CAS Center for Excellence in Molecular Cell Science, Institute of Biochemistry and Cell Biology, Chinese Academy of Sciences.

Note that full information on the approval of the study protocol must also be provided in the manuscript.

Plants

Seed stocks	N/A
Novel plant genotypes	N/A
Authentication	N/A
